# Albumin infusion in spontaneous bacterial peritonitis: another brick off the wall?

**DOI:** 10.1186/s13613-018-0450-2

**Published:** 2018-10-29

**Authors:** Damien Roux, Richard Moreau, Didier Dreyfuss

**Affiliations:** 1 Intensive Care Unit, Louis Mourier Hospital, AP-HP, 178 rue des renouillers, 92700 Colombes, France; 2 Sorbonne Paris Cité, Univ Paris Diderot, Paris, France; 3 IAME, UMR 1137, INSERM, Paris, France; 4 Centre de Recherche sur l’inflammation (CRI), U1149, INSERM, Paris, France; 5 Département Hospitalo-Universitaire (DHU) UNITY, Service d’Hépatologie, Hôpital Beaujon, AP-HP, Clichy, France; 6 Laboratoire d’Excellence (Labex) Inflamex, CUE Sorbonne Paris Cité, Paris, France

## Main text

Albumin infusion did not convey any survival benefit compared to normal saline in a randomized controlled study (RCT) which involved a very large population of ICU patients who required intravascular fluid resuscitation [1]. There was, however, a nonsignificant trend in favor of albumin in patients with severe sepsis [2]. This trend was contradicted in another RCT which did not show any difference in mortality when albumin was added to normal saline during severe sepsis or septic shock compared with saline alone [3]. Post hoc analyses, with their inherent limitation, suggested a trend for higher mortality in patients with severe sepsis without shock who received albumin and a significant reduction in mortality with albumin in patients with septic shock [3]. A conservative conclusion of all these studies is the lack of strong evidence in favor of albumin during sepsis without shock.

Spontaneous bacterial peritonitis (SBP) in cirrhotic patients is a frequent septic condition which carries an important morbidity and mortality. Patients often present all the characteristics indicating severe sepsis which would make them eligible for the above-mentioned studies [1, 3]. However, they were excluded from the ALBIOS study [3] and no detail on this specific patient population was given in the SAFE study [1, 2]. Therefore, there are no data from studies on sepsis that could suggest a potential advantage of albumin infusion over normal saline during SBP (see Table 1). Given all the above, one should not expect any major positive effect. Despite this lack of solid evidence, all guidelines on the treatment of SBP mandate the infusion of albumin in addition to antibiotics, at least for patients at risk of acute kidney injury [4–6]. In fact, this recommendation is disputable for three reasons:

1. Guidelines, even if some are posterior, do not take the above-mentioned studies into account.
2. This recommendation is based on RCTs that did not respect clinical equipoise, that is, “a genuine uncertainty within the expert clinical community, not necessarily on the part of the individual investigator regarding the comparative therapeutic merits of each arm in a trial” [7]. This is an ethical prerequisite for the scientific value of a clinical trial [7].
3. Indeed, as detailed in the princeps article by Sort et al. [8] and in a meta-analysis by Salerno et al. [9] which integrates all RCTs on albumin infusion during SBP and constitutes the cornerstone of recommendations in favor of albumin, these RCTs compared vascular filling with albumin with the absence of any intravascular fluids [8, 10, 11] or a hydroxyethyl starch infusion which was subsequently shown to be nephrotoxic [12] (Table 1). One may wonder whether such studies would get published nowadays. The only conclusion that can be drawn from these studies is the confirmation of the paramount importance of fluid resuscitation during sepsis, at least at the initial phase. In addition, most recommendations were written by experts who had financial ties with the manufacturers of albumin solutions. Many major journals now preclude that guidelines be written by authors with such ties [13]. Interestingly, the only recommendation published by authors without financial ties highlighted that the previous studies which were selected for the meta-analysis by Salerno et al. [9] could be “criticized as the control groups were not given an equivalent amount of fluid as crystalloid” and added that “further studies are required before making any formal recommendations about the use of albumin in SBP” [6].

**Table 1:** Characteristics, details of interventions used and outcomes measured in randomized trials studying albumin treatment during spontaneous bacterial peritonitis, sepsis other than SBP in cirrhotic patients and general ICU population with sepsis

Trial	*N*	Age, *y*^a^	Experimental treatment	Control treatment	Mortality (albumin vs. control group; *p*)
*Spontaneous bacterial peritonitis*					
Sort et al. [8]	126	61.0 (7.9)	20% albumin	No vascular filling	Favors albumin (22% vs. 41%; *p* = 0.03)^b^
Xue et al. [10]	112	22–70	20% albumin	No vascular filling	Favors albumin (10% vs. 34%; *p* = 0.002)^c^
Fernandez et al. [14]	20	61.0 (9.5)	20% albumin	6% HES 200/0.5	NS (not significant) (0% vs. 20%; *p* = 0.47)^c^
Chen et al. [11]	30	56.5 (11.5)	20% albumin	No vascular filling	NS (26.7% vs. 40%; *p* = 0.70)^c^
*Sepsis other than SBP in cirrhotic patients (no septic shock)*					
Guevara et al. [15]	97	56 (11)	20% albumin	No vascular filling	NS (17% vs. 20%; *p* = 0.78)^b^
Thévenot et al. [16]	193	55.3 (8.6)	20% albumin	No vascular filling	NS (30% vs. 22%; *p* = 0,16)^b^
*Sepsis and septic shock in general ICU population* ^d^					
SAFE study [2]^e^	1218	60.5 (17.2)	4% albumin	NaCl 0.9%	NS (30.7% vs. 35.3%; *p* = 0.09)^f^
ALBIOS study [3]	1810	69 [59–77]	20% albumin	Crystalloids	NS (20.9% vs. 21.1%; *p* = 0.87)^f^

^a^Mean (SD) or median [IQ] or range

^b^3-month mortality

^c^Hospital mortality

^d^Only studies including more than 100 patients are presented

^e^Predefined subgroup of patients with severe sepsis from the SAFE study

^f^28-day mortality

It stems from the preceding that superiority of albumin over normal saline has never been proved or even tested during SBP. This is not a trivial issue when one keeps in mind that the cost of infused albumin in protocols for SBP treatment amounts to about 300 euros for each individual patient. Given the incidence of SBP, million euros (or dollars) could be saved if normal saline proved at least as effective as albumin.

Overall, there is abundant evidence that recommendations for albumin during SBP have to be challenged by trials using much cheaper alternatives such as saline solution as comparator.

We are currently planning to undergo such a trial for which we asked a grant from French Ministry of Health. This study might be dedicated to Roger Waters (with salt) and may remove another brick off the wall of recommendations for albumin.

## Abbreviations

ICU: intensive care unit
RCT: randomized controlled study
SBP: spontaneous bacterial peritonitis
